# Supplementary material for: Intermolecular-Type Conical Intersections in Benzene Dimer
Source: Int J Mol Sci. 2023 Feb 2;24(3):2906. doi: 10.3390/ijms24032906 (PMC9917476; doi:10.3390/ijms24032906)
Supplement: Supplementary file 1 [file ijms-24-02906-s001.zip › ijms-2175483-supplementary.pdf]

Supplementary Materials: Intermolecular-type conical intersections in benzene dimer

Attila Bende [1,*] and Alex-Adrian Farcaş [1]

L1: Geometry structures:

Structure name	: $R_e(S_0)$
Method	: DFT (ωB97X-D3/ma-def2-TZVPP)

24

C	0.16755025682620	0.15803578810550	-0.38459061997770
C	1.51321766139978	0.01079268527048	-0.68819018423892
C	2.28047103304959	1.12399429819786	-0.99722345909495
C	1.70115805111480	2.38501349554715	-1.00741234610902
C	0.35512893389640	2.53099563530205	-0.70738639823690
C	-0.41130131535660	1.41779131257521	-0.39464913236115
H	-0.42863917334644	-0.70863260625668	-0.12874159861690
H	1.96642113310338	-0.97265667319859	-0.67802529543420
H	3.33173352859505	1.00882305179108	-1.23032029448633
H	2.30011591162734	3.25417567180729	-1.24928066232854
H	-0.09724090928484	3.51476439355759	-0.70986578842554
H	-1.45849251299371	1.53424212385561	-0.14647668891147
C	-0.37836887395182	0.42410237858500	3.39121431458521
C	0.97086834518298	0.28852463665143	3.10032523877149
C	1.73056191978806	1.40726198887051	2.79156421374091
C	1.14126785009880	2.66224355193723	2.77723504533895
C	-0.20781496672770	2.79899849048810	3.07010073990427
C	-0.96836833266284	1.67996784217166	3.37485584243150
H	-0.97191969076266	-0.45004074415075	3.62845879719222
H	1.42984961683168	-0.69217849529421	3.10617452433948
H	2.78031915756564	1.29971744601705	2.55039706645812
H	1.73272725557698	3.53403552633335	2.52779903303920
H	-0.66785575279843	3.77920403316131	3.05506285112859
H	-2.02247912677167	1.78576416867477	3.59932480129168

Structure name	: $R_e^{CAo}(S_0)$
Method	: DFT (ωB97X-D3/ma-def2-TZVPP)

24

C	0.36464760085564	0.25709494423586	0.35338463452549
C	1.86863920585243	0.28983980309898	-0.03363782207107
C	2.27200194631667	1.51795097569305	-0.79029133175789
C	1.38313929258104	2.36224996919631	-1.30633809788770
C	-0.06214603163764	2.15712852981624	-1.15272133006938
C	-0.54645867132677	1.17804023992195	-0.39358407812151
H	-0.02369742271804	-0.76168680439294	0.31415763835586
H	2.22710920541987	-0.58908996576593	-0.57675289929900
H	3.33246483768361	1.68848569314746	-0.93756250772157
H	1.71921244459674	3.22442377216316	-1.86935780289960

H	-0.73313091000219	2.82424893116364	-1.68005558080004
H	-1.61693674275895	1.04012942672144	-0.29000919720837
C	0.74838227961066	0.60367889161557	1.82814702202831
C	2.22522194244262	0.25771274398299	1.48860973004525
C	3.27450009864166	1.18811284446720	2.00679592626981
C	2.95337600093569	2.38555435201422	2.48900322309763
C	1.55729053438578	2.83326508485694	2.55539527469454
C	0.54509159129731	2.02643874700255	2.24980692468655
H	0.27358490334163	-0.05304820801413	2.56237006613297
H	2.46077446245239	-0.76732343189059	1.77850111792928
H	4.31054552217546	0.87087910579154	1.96623491590172
H	3.72441535736867	3.05656581009621	2.84743460036542
H	1.36067590404949	3.84900465165916	2.87584618369861
H	-0.47566335156387	2.38357389341903	2.32639339010466

Structure name : $R_e^{CAa}(S_0)$
Method : DFT (ωB97X-D3/ma-def2-TZVPP)

24

C	0.62349266032939	1.04848048820938	0.45221050850223
C	1.98551547668296	0.95170452076915	-0.28909537605197
C	1.92035972686812	1.37815836893285	-1.72252114470336
C	0.76778073882353	1.58249474687725	-2.35678288456268
C	-0.52734224529367	1.38687551138570	-1.69401749392035
C	-0.60542214953929	1.11435081379201	-0.39309877633831
H	0.53501418130170	0.27256255923848	1.21331849466634
H	2.48814990025958	-0.01756203060718	-0.21555737621273
H	2.86225323789522	1.49891321921555	-2.24782941770557
H	0.76906811074960	1.87908442686991	-3.39868284800141
H	-1.42621542016544	1.45897504771047	-2.29402547153635
H	-1.56856425771064	0.96325639953332	0.08120780024997
C	1.11166546628294	2.37550814676019	1.11500583574695
C	2.56057191108348	2.02782501295336	0.67375178827746
C	3.40700986935255	1.47433000579984	1.77667051212875
C	3.02992262863145	1.50494214109049	3.05309144867269
C	1.75863009943133	2.10764681721037	3.47172212608351
C	0.86954494984773	2.53896130471013	2.57946828544307
H	0.72454212520923	3.22740437824639	0.55512633934242
H	3.09786299465900	2.83301031054645	0.16337942799260
H	4.36830619443964	1.05343135951333	1.50001856779974
H	3.68118443305567	1.10108876260381	3.81875521199738
H	1.56370289130222	2.20608367794179	4.53258155174600
H	-0.06045352349631	2.99173401069695	2.90419289038361

Structure name : $R_e(S_1)$
Method : TDDFT (ωB97X-D3/ma-def2-TZVPP)

24

C	0.16622940899554	0.17630213654732	-0.33841170731140
C	1.52937251001468	0.02668323359299	-0.62351120592893

C	2.30580100460586	1.14812544154930	-0.94355006957887
C	1.71949508625313	2.41985033112146	-0.97155085014310
C	0.35726155342980	2.56966666333839	-0.68274737853937
C	-0.41869166383757	1.44897251203894	-0.36076423665417
H	-0.43324331265816	-0.69090754818253	-0.09669971037958
H	1.97879860508356	-0.95699585946752	-0.60883424046735
H	3.35996451064180	1.03454796904632	-1.15755599754154
H	2.31927655764841	3.28842861941345	-1.20829503783134
H	-0.09069021333284	3.55424249460922	-0.69394590867408
H	-1.46715334339926	1.56728763583715	-0.12268759316184
C	0.81620156664441	0.58804197784603	2.63759318469234
C	2.17938602549572	0.43513975258483	2.35570204391666
C	2.95926386063114	1.55410913767761	2.03716099257123
C	2.37534652269422	2.82710603490417	2.00264197271154
C	1.00912429909391	2.97842179155463	2.27200216682489
C	0.22917424025855	1.85892686508372	2.59054911940788
H	0.21468704274542	-0.27828277142094	2.87826062310655
H	2.62671770800288	-0.54955001087090	2.37664305375215
H	4.01065756814541	1.43469366404002	1.81299809399448
H	2.97757547818380	3.69300250478732	1.76302925732764
H	0.55843007971822	3.96109200118884	2.23975078617623
H	-0.82699509505881	1.97431542318001	2.79334264172987

Structure name : CI_M(I)
Method : Spin-Flipped TDDFT (ωB97X-D3/ma-def2-TZVPP)

24

C	0.00596198687068	0.01481339486688	0.01355896097355
C	1.45983752665012	0.02133601227033	0.01357228331248
C	2.24008024689950	1.23128669976950	0.01195823290366
C	1.75715985649155	1.15253338234636	-1.34267965242936
C	0.38402043583304	1.45444172450415	-1.71335933562192
C	-0.56953759483572	0.88011007196045	-0.89487572193644
H	-0.54288621609919	-0.65520048976291	0.66000819615705
H	1.97371428192024	-0.91808252676084	0.21193152962639
H	-1.63157368374774	1.05685122418778	-0.97671163150983
H	0.16739422189068	2.04011761149615	-2.59590564067453
H	3.30108110830546	1.19506776163872	0.21654591475747
H	2.48898590347725	1.04891652486912	-2.14228461881915
C	-2.48236376881904	2.27498050085674	2.05627642258635
C	-1.34277893413780	1.98112836289731	2.79223449576070
C	-2.44727984416542	3.27455036562633	1.09529162269198
C	-0.16961700120288	2.68495662521633	2.56602711283854
C	-1.27198157866521	3.97685709001412	0.86771892853012
C	-0.13362717519947	3.68189445354862	1.60224269085277
H	-3.39738568607924	1.72246165605456	2.23085895094886
H	-1.36869689201318	1.19824088158781	3.54017906007993
H	-3.33509902394590	3.50372983616035	0.51867645010188
H	-1.24189777821572	4.75072539983340	0.11087612394217
H	0.72314272904434	2.44971559217275	3.13094110868747
H	0.78813478905583	4.21670952766117	1.41449691630325

Structure name : CI_M(II)
Method : Spin-Flipped TDDFT (ωB97X-D3/ma-def2-TZVPP)

24

C	-0.00007916868423	-0.00206546527766	0.00137089805298
C	1.45817086966759	-0.00165524047606	0.00196986547458
C	2.24364699504036	1.20224851014770	0.00303015689612
C	1.76005634853262	1.13214350959778	-1.35183087908222
C	0.39011030778902	1.44870864474089	-1.71209928262231
C	-0.56911821881030	0.87668680733120	-0.89428831764456
H	-0.55118657202270	-0.69601117624334	0.62063818887562
H	1.96684897654164	-0.94402930935660	0.19702675700233
H	-1.63145107651801	1.04455851735180	-0.99248974689819
H	0.17538896310631	2.03788043773317	-2.59268338936055
H	3.30465217647777	1.15770683937013	0.20633117313216
H	2.48522459376820	1.02576829323144	-2.15709027196592
C	-1.97081940511812	-1.85265232680383	-3.10604140772480
C	-1.00036032198164	-2.55990586753644	-2.41152022039325
C	-1.60353823742479	-1.00573055560802	-4.14174377834211
C	0.33678726887378	-2.42076124639072	-2.75295606121374
C	-0.26607497885486	-0.86730798326312	-4.48327830053984
C	0.70416812596328	-1.57430350413904	-3.78802643681597
H	-3.01451363757669	-1.95976518370150	-2.83820324716375
H	-1.28648271572929	-3.21763489088536	-1.60031445110005
H	-2.36055099602540	-0.45232897086943	-4.68335934668984
H	0.02139870839147	-0.20740096348782	-5.29236053206173
H	1.09492646906215	-2.97045172084805	-2.20941961350811
H	1.74830962679293	-1.46475273424659	-4.05282008381879

Structure name : CI_D(I)
Method : Spin-Flipped TDDFT (ωB97X-D3/ma-def2-TZVPP)

24

C	0.00000000000000	0.00000000000000	0.00000000000000
C	1.36340728835100	0.00000000000000	0.00000000000000
C	2.13006891675498	1.22952755365549	0.00000000000000
C	1.28706450040853	2.45907980493519	-0.05582543810145
C	-0.10442913447795	2.38684789237767	-0.18948865847330
C	-0.76628741758924	1.19097442523719	-0.07970146991832
H	-0.51709276861129	-0.95277293974291	0.01221386158665
H	1.89648081742982	-0.94170189425762	-0.00771830436942
H	3.02003533117809	1.21171139055230	-0.62942189561609
H	1.78570774448894	3.38659067090957	-0.31073482564585
H	-0.66033407718539	3.31028701932341	-0.30479770510427
H	-1.84498672390497	1.14162980860596	-0.12572580137826
C	2.21659457320089	-0.18921053141712	3.04606305699835
C	3.20602291073909	0.40710182725281	2.32959473102381
C	2.94157242742521	1.61585411679398	1.60977618617251
C	1.75547904345950	2.42310396812768	1.99511312909028
C	0.77724822376597	1.77545499320632	2.79069896018293
C	0.98152511978734	0.49859060715682	3.24063804311379
H	2.36793535591452	-1.15412446953527	3.51040869508410

H	4.15845592410407	-0.08785713919771	2.18445224218314
H	3.80822917908783	2.19082646120967	1.29972018323869
H	1.82698002253890	3.50194363013243	2.03627449765691
H	-0.12920321994404	2.30302995895289	3.05556796605726
H	0.20889559729960	0.02283690401248	3.83264263554284

Structure name : CI_D(II)
Method : Spin-Flipped TDDFT (ωB97X-D3/ma-def2-TZVPP)

24

C	0.00005190330919	0.00007491260228	0.00026109390845
C	1.40605404235885	0.00008614283218	0.00004194268944
C	2.13278181747219	1.29165288475398	-0.00015600864045
C	1.59249725506510	1.56605990689099	-1.34929395909115
C	0.20894811250938	1.76080073128865	-1.51389124785649
C	-0.64534408016339	1.04036314843342	-0.67580786663911
H	-0.54642274289843	-0.83719314063071	0.41603959080334
H	1.97293096383414	-0.91853365790680	0.08930874621700
H	2.28185306996173	1.70945980235651	-2.17289574654743
H	-0.16879913580163	2.36502458775208	-2.32941343230993
H	1.76934995311274	2.32054257515940	1.14885355356775
H	2.20189298494381	1.79695094754068	2.46631345404630
C	3.07904157792147	2.46113619736838	3.26452461277872
C	3.60679451607582	3.71132669829838	2.88816244511445
C	3.20905690736491	4.28860948224141	1.66578697239845
C	2.33487297848508	3.65519833858936	0.84224685859376
C	1.81086602565433	0.83343943411630	2.77261227023729
C	4.30913696864838	4.22514579889950	3.52995787309765
H	-1.71907633320397	1.15643269324912	-0.68711272889699
H	2.04373398911238	4.11058279549397	-0.09713398872124
H	3.60879189557837	5.25495343758804	1.38248706488184
H	3.37845382571071	2.02358887410323	4.20957279757088
H	0.66884571250100	2.36431199390176	1.10124748234944
H	3.21040118739265	1.14845030174534	-0.01923878865173

Structure name : CI_D(60°)
Method : Spin-Flipped TDDFT (ωB97X-D3/ma-def2-TZVPP)

24

C	0.000000	0.000000	0.000000
C	1.415415	0.000000	0.000000
C	2.157884	1.291137	0.000000
C	1.337057	2.467924	0.020636
C	-0.023338	2.389635	0.164697
C	-0.718494	1.153757	0.173458
H	-0.511441	-0.953456	-0.069694
H	1.941574	-0.872275	-0.364170
H	3.060097	1.350001	-0.598919
H	1.815118	3.435797	-0.067248
H	-0.595991	3.309206	0.206136
H	-1.797499	1.136786	0.234005

C	2.963878	0.994547	1.728445
C	4.300342	0.534753	1.594354
C	4.621319	-0.794381	1.663177
C	3.635295	-1.727206	2.088643
C	2.338080	-1.357463	2.290727
C	1.921681	-0.033527	1.952022
H	2.824438	1.961066	2.198362
H	5.078376	1.266420	1.406606
H	5.638792	-1.124891	1.505261
H	3.935444	-2.748917	2.288889
H	1.605210	-2.073645	2.638843
H	0.958536	0.317755	2.299287

Structure name : CI_D(100°)
Method : Spin-Flipped TDDFT (ωB97X-D3/ma-def2-TZVPP)

24

C	0.00000001138510	-0.00000000713253	0.00000000301917
C	1.36551251444876	-0.00000000013331	-0.00000000324675
C	2.25065738778407	1.16669274460386	-0.00000000459957
C	1.32936446717755	2.39569079860773	-0.12322170389376
C	-0.07305326764585	2.33566926734850	-0.22128207344734
C	-0.78131213540826	1.16970235460918	-0.06002940710196
H	-0.48964144404116	-0.96800701304085	-0.00208239366212
H	1.84851913499835	-0.97102053494299	-0.04398065232195
H	3.13497079487939	1.09809497729647	-0.63151445109800
H	1.80874298436257	3.34093768933971	-0.35557995149115
H	-0.59611620479980	3.28231758970403	-0.31178218304325
H	-1.85998754401765	1.13910178051568	-0.08035799444499
C	2.82633558321348	1.79385231577796	1.53243237346062
C	3.54414639364823	0.70585598653318	2.11572290002965
C	2.77525049515290	-0.19642729976925	2.78991397804047
C	1.39431528895112	0.11568683976071	3.01433313463678
C	0.78415427882259	1.30154045491283	2.68813335097894
C	1.51537340348054	2.26613319435439	1.95927261002071
H	3.46545578960007	2.60366084734349	1.18401789770251
H	4.58240422811957	0.53498614939741	1.86659429495899
H	3.17009377771737	-1.12863143084997	3.16981466027833
H	0.81151928837962	-0.61057885425348	3.57037904601284
H	-0.22678651391698	1.49948199647416	3.01436946442047
H	1.32352335774762	3.31884194018215	2.11400079662865

Structure name : TS_D(I)
Method : NEB-DFT (ωB97X-D3/ma-def2-TZVPP)

24

C	-0.51721208146803	-1.51686488978709	-1.42005565939467
C	0.73460782889479	-0.99062223805334	-1.56367044044857
C	0.92807059883376	0.43254194210519	-1.46870467262915
C	-0.24041098138317	1.26792471681802	-1.53532974034362
C	-1.48068053444165	0.71810961279280	-1.38177213789464

C	-1.62659791460993	-0.67316338072253	-1.22142855702544
H	-0.66022802463714	-2.58971548719568	-1.44417244341329
H	1.58716371882948	-1.63295235267586	-1.74146531685160
H	-0.12486615108030	2.33439539608350	-1.67810406076736
H	-2.35740573557881	1.35299439802511	-1.37407724451161
H	-0.70421553625095	0.96387627428466	1.77749648580986
H	-0.80449468382067	-0.43002001924943	1.95295848586536
C	0.32420275760487	-1.24285560058112	1.73336459757530
C	1.45222546736316	-0.71922138017398	1.16956075801596
C	1.47550182012400	0.65783189119856	0.75470545302208
C	0.41581702141555	1.51170788308971	1.22157687605342
C	-1.68732986270725	-0.84983182963743	2.41758405531711
C	2.32764758871587	-1.33601085361805	1.01384679904394
H	-2.61550232763301	-1.10108117125185	-1.11821018327134
H	0.50745993503288	2.58401128864705	1.10916055251352
H	2.43075575833170	1.10643171671549	0.51264591574088
H	0.28826201618720	-2.29132269697101	2.00012567594463
H	-1.52852460586156	1.59898785962620	2.07598193384624
H	1.87575392813920	0.84484892053108	-1.79201713219701

Structure name : CI_D(I)
Method : CASSCF(12,11)/6−31G**

24

C	-1.2588150077	-0.9562743753	-1.3275134759
C	-0.2898153954	-1.5477864249	-0.5822507950
C	1.0459415608	-0.9529657676	-0.4786998367
C	1.2901221179	0.1950899178	-1.3785311640
C	0.2472755508	0.6946655431	-2.2126471673
C	-1.0325151696	0.2345884522	-2.1163296321
H	-2.2214502194	-1.4374229995	-1.3863068682
H	-0.4790911146	-2.4783931981	-0.0811517831
H	1.8528077451	-1.6475115319	-0.3324762004
H	2.3121976983	0.3854957891	-1.6586147363
H	0.4788594925	1.5232676811	-2.8619338193
H	-1.8336575640	0.6504548152	-2.6983717098
C	-1.0325788974	-0.2435703799	2.1036916310
C	0.2465360113	-0.7060398778	2.1993297130
C	1.2886431653	-0.1993934481	1.3684120729
C	1.0298812773	0.9712473240	0.4993111779
C	-0.2964448768	1.5544815767	0.5971472669
C	-1.2532018811	0.9572398989	1.3309150817
H	-1.8383824413	-0.6650283658	2.6742211410
H	0.4734551665	-1.5421090736	2.8400069709
H	2.3105779811	-0.3714726788	1.6599105469
H	1.8378959976	1.6571516287	0.3295593548
H	-0.4949178943	2.4801285375	0.0915390015
H	-2.2198881751	1.4301573610	1.3898366746

Structure name : CI_D(II)
Method : CASSCF(12,11)/6−31G**

24

C	-0.3165472354	-1.1776541971	-2.5020904796
C	0.8444495090	-1.0490355039	-1.6500466485
C	0.8557189543	0.0050114516	-0.5869070923
C	0.8415837403	1.0517647613	-1.6497437492
C	-0.3127323057	1.1745465975	-2.5101639667
C	-0.9865451800	-0.0033472547	-2.8587719550
H	-0.5956624160	-2.1368183419	-2.9009659748
H	1.7016203226	-1.6824932877	-1.7910310192
H	1.7035664557	1.6759595261	-1.8039131649
H	-0.5746631450	2.1270149231	-2.9357341724
H	-0.2764470530	-0.0003867480	0.4657154464
H	-0.2127832589	-1.2512564779	1.3077032477
C	-0.0997419734	-1.2279671046	2.6680921675
C	-0.0414805855	0.0007033438	3.3877646410
C	-0.1050293706	1.2283608671	2.6682506652
C	-0.2193798720	1.2506711095	1.3074793784
C	-0.2561238994	-2.1936269646	0.7898533442
C	0.0493428786	0.0008575245	4.4579953845
H	-1.8865055197	-0.0042522324	-3.4455737155
H	-0.2656978431	2.1926641815	0.7891772153
H	-0.0638273457	2.1562687488	3.2130038985
H	-0.0551793999	-2.1554417405	3.2133229863
H	-1.2203035196	-0.0032520285	-0.0781700862
H	1.8082296004	0.0063075263	-0.0713910447

L2: Molecular orbitals for CI_D(I):

Occupied orbitals

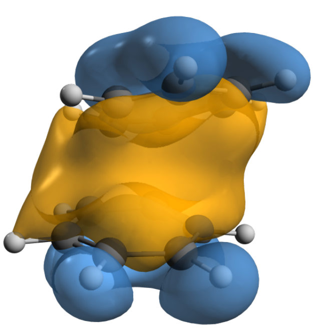
MO: 33

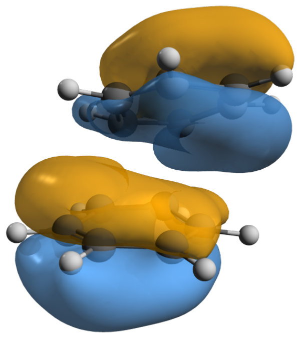
MO: 38

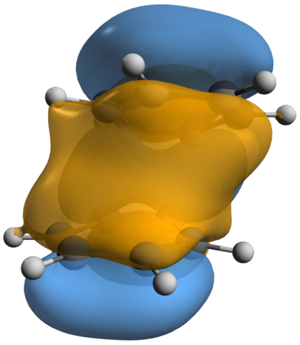
MO: 39

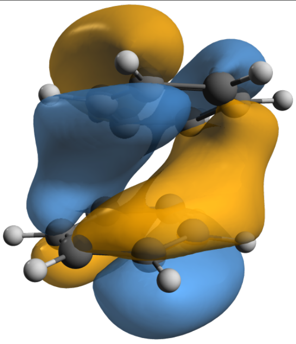
MO: 40

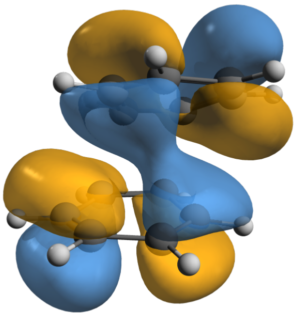
MO: 41

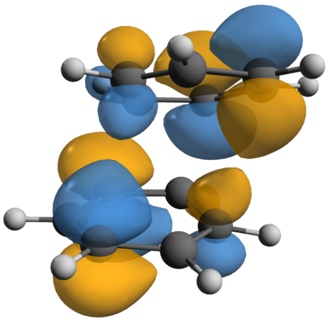
MO: 42

Virtual orbitals

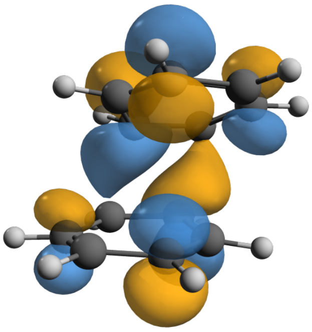
MO: 43

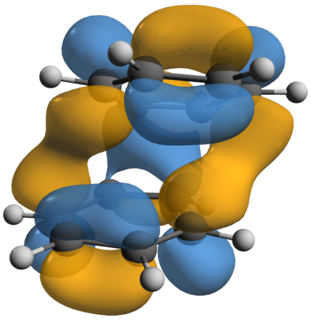
MO: 44

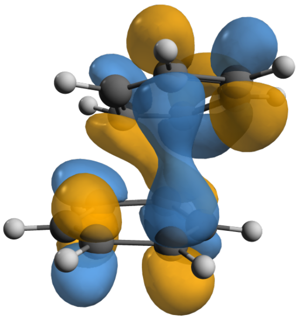
MO: 45

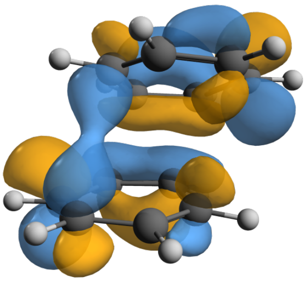
MO: 47

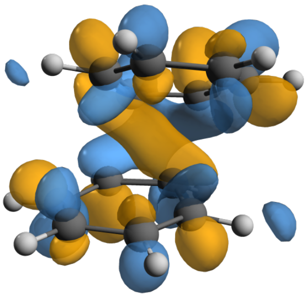
MO: 60

L3: Molecular orbitals for CI_D(II):

Occupied orbitals		
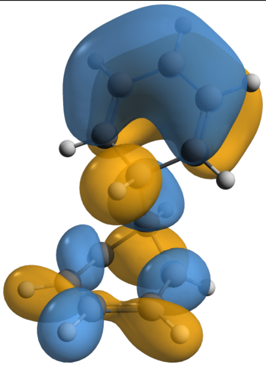 MO: 36	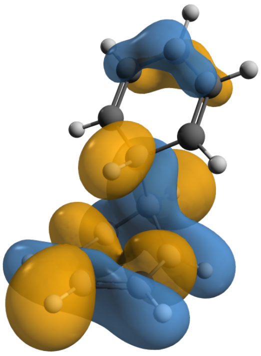 MO: 38	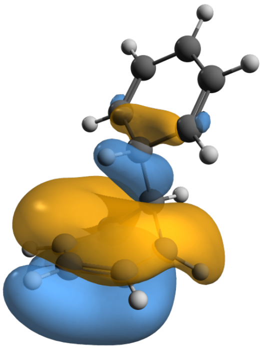 MO: 39
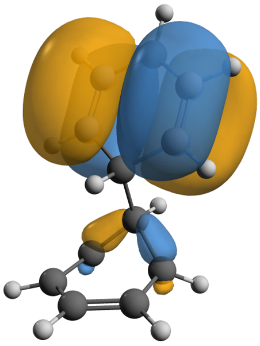 MO: 40	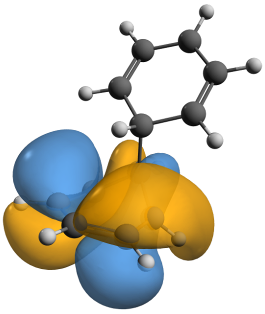 MO: 41	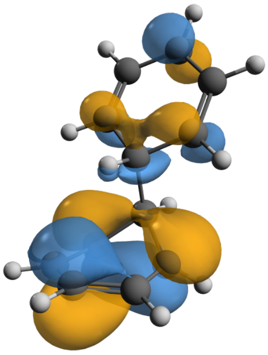 MO: 42
**Virtual orbitals**		
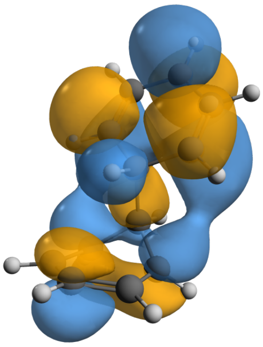 MO: 43	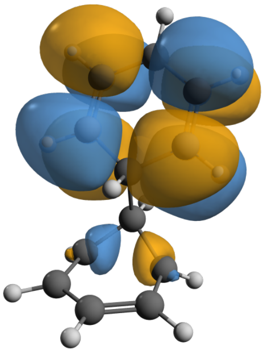 MO: 44	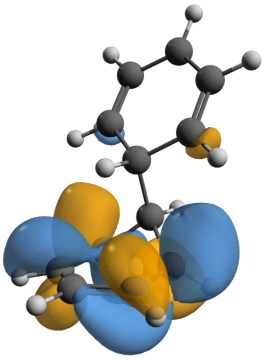 MO: 46
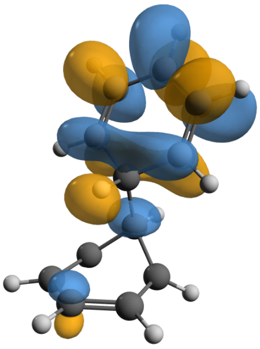 MO: 47	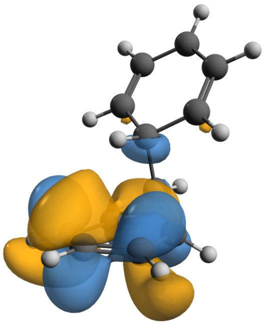 MO: 51	

Table S1. The bond lengths between the carbon atoms (in Å) of the half-boat geometry configurations computed for different conical intersection (CI) geometries at SF-TDDFT/ωB97X-D3/ma-def2-TZVPP level of theory.

Geom.	Benzene half-boat monomer's C–C bonds						
	C_1-C_2	C_2-C_3	C_3-C_4	C_4-C_5	C_5-C_6	C_1-C_6	C_2-C_4
Mon.	1.452	1.443	1.443	1.452	1.381	1.381	1.789
CI_M(I)	1.454	1.440	1.440	1.454	1.381	1.381	1.791
CI_M(II)	1.458	1.437	1.440	1.451	1.384	1.378	1.791
